# Molecular Characterization of Local Walnut (*Juglans regia*) Genotypes in the North-East Parnon Mountain Region of Greece

**DOI:** 10.3390/ijms242417230

**Published:** 2023-12-07

**Authors:** Ioannis Manthos, Thomas Sotiropoulos, Lefkothea Karapetsi, Ioannis Ganopoulos, Emmanouil D. Pratsinakis, Eleni Maloupa, Panagiotis Madesis

**Affiliations:** 1 Department of Nut Trees, Institute of Plant Breeding & Genetic Resources, Hellenic Agricultural Organization (ELGO)-DIMITRA, Neo Krikello, 35100 Lamia, Greece; jmanthos@elgo.gr; 2 Department of Deciduous Fruit Trees, Institute of Plant Breeding & Genetic Resources, Hellenic Agricultural Organization (ELGO)-DIMITRA, 59200 Naousa, Greece; thsotiropoulos@elgo.gr; 3 Laboratory of Molecular Biology of Plants, School of Agricultural Sciences, University of Thessaly, 38446 Volos, Greece; lefki8@certh.gr; 4 Institute of Applied Biosciences, Centre for Research & Technology-Hellas, 57001 Thessaloniki, Greece; epratsina@certh.gr; 5 Institute of Plant Breeding and Genetic Resources, Hellenic Agricultural Organization Dimitra, 57001 Thessaloniki, Greece; iganopoulos@elgo.gr (I.G.); emaloupa@elgo.gr (E.M.)

**Keywords:** *Juglans regia*, local walnut genotypes, molecular characterization, SSR

## Abstract

Walnut is one of the most important nuts regarding their production and consumption. The available but uncharacterized genetic resources of walnut are important for the development and breeding of local varieties. Greece holds an important number of genetically uncharacterized walnut landraces, especially within the area of Parnon, which is considered to play a significant role as an in situ gene bank, due to its unique location traits. However, the genetic characterization and further use of these resources has been insufficient, due to the absence of genetic studies. In this study, we implemented SSR molecular markers, both to genetically characterize the walnut tree genetic diversity of the Parnon area and to identify its unique genetic structure, which will form the starting material for subsequent breeding programs. Overall, high levels of genetic variation were found among the individual walnut accessions that were collected in the Parnon mountain region.

## 1. Introduction

One of the most economically significant cultivated nut tree species is the Persian walnut (*Juglans regia* L.). Walnut cultivation is distributed worldwide, and global walnut production has been steadily increasing during the last decade (2012–2021) from 2,368,722 to 3,500,172 tn [1]. Numerous studies and reviews have highlighted walnuts’ nutritive value because of their rich content of a variety of nutrients and phytochemical agents important to human health [2,3,4,5,6].

Greece, according to FAOSTAT, held the fourth position in Europe concerning the average walnut production from 2012 to 2021 [1]. Moreover, specifically for the year 2021, Greece held the first position in Europe and the ninth position worldwide, revealing a significant and steady increase from 23,432 tn to 62,810 tn during the last decade [1]. These figures reveal the high interest and potential of walnut cultivation in Greece.

Greece’s walnut cultivation is characterized by two different cultivating systems. On one hand, there are the new, steadily increasing walnut orchards, in which selected universal cultivars are chosen for cultivation. The most frequently used cultivars are the lateral bearing “Chandler”, “Lara”, and “Pedro” and the apical bearing cultivars “Franquette” and “Ronde de Montignac”. On the other hand, many traditional native landraces still exist. These landraces have a long history of cultivation, are well adapted to the region’s climate, are productive, and support rural economy.

In the frame of European projects, local landraces have been located and characterized in various regions of Greece. The majority of them are located in the regions of Central Macedonia, Thessaly, Peloponnese, and Crete [7,8,9]. Some of these landraces, along with other internationally cultivated walnut genotypes, were kept in gene banks and were further evaluated for their genetic diversity. The results revealed that the examined Greek walnut landraces presented higher genetic diversity, compared to common walnut cultivars [10].

Walnut cultivation used to be very common in the mountainous Greek villages. Walnut trees were planted in almost every house yard, for their delicious fruits and the thick shade they provided. Walnut tree fruits and leaves were exploited for their pharmaceutical uses, and also to make hair and textile dyes [11].

Peloponnese is known as one of the most favorable areas for walnut cultivation, and it accounts for the second highest walnut production in Greece [12]. It is famous for its thumping mountain ranges, where walnut cultivation still relies on old landraces, and specifically in the Parnon mountain range, which extends into the prefectures of Laconia and Arcadia.

Although an effort was made to identify, select, and use Greek walnut landraces in the past, studies on their genetic diversity were not systematic. Nowadays, in the Parnon region, where a vast gene pool of local walnut landraces can still be found, new efforts are being made to characterize and exploit the local walnut population.

Various methods have been employed to investigate the genetic diversity and relationships among Persian walnut cultivars, which include amplified fragment-length polymorphism (AFLP) [13], randomly amplified polymorphic DNA (RAPD) [14], and inter-simple sequence repeat (ISSR) markers [10,15,16]. The use of molecular markers underlines the importance of precise cultivar identification, establishing genetic fingerprints, and verifying parentage and information on genetic relationships.

Simple Sequence Repeat (SSR) molecular markers are considered to be a well-established and effective approach for evaluating the genetic diversity of *Juglans regia* (common walnut) cultivars and have been widely adopted in recent research studies that primarily aimed to characterize and discern walnut cultivars based on their distinct genetic profiles [17,18,19,20,21,22]. The estimation of various diversity metrics, including the polymorphism information content (PIC), the observed heterozygosity, and the expected heterozygosity, provides a quantitative measure of genetic diversity [22]. Furthermore, SSR markers could play an essential role in genetic mapping investigations aiming to pinpoint quantitative trait loci (QTLs) linked to important walnut traits, such as disease resistance, nut quality, and yield [23,24]. Such results can then be implemented in marker-assisted selection (MAS) strategies in breeding programs.

SSR markers are characterized by their high polymorphism, indicative of the presence of multiple alleles or genetic variations within a given population. Their polymorphic nature empowers them to accurately discriminate between genotypes, rendering them invaluable in genetic diversity evaluations and genetic mapping efforts. Given their property of co-dominant inheritance, SSR markers can effectively differentiate heterozygous individuals (bearing two differing alleles) from homozygous individuals (possessing two identical alleles), a characteristic that contributes to precise genotype identification and the comprehensive exploration of genetic diversity [25]. Moreover, the variable count of repeat units within microsatellites enables discrimination among closely related individuals, an assessment of genetic diversity, and differentiation between various cultivars or breeding lines [26].

Capillary electrophoresis (CE) has significantly advanced the field of genotyping studies by offering a highly efficient, precise, and automated method for separating and analyzing DNA fragments. Capillary electrophoresis provides excellent separation of DNA fragments based on their size, making it possible to distinguish between alleles that may differ by just a few base pairs. This high resolution is crucial for accurately genotyping genetic variations, such as single-nucleotide polymorphisms (SNPs) and microsatellites (SSRs). Overall, capillary electrophoresis has revolutionized genotyping studies by offering an efficient, automated, and high-resolution method for DNA fragment analysis, making it a valuable tool for various scientific applications, including genotyping, proteomics, and environmental monitoring [27,28,29].

Using capillary electrophoresis (CE) with Simple Sequence Repeat (SSR) markers for genotyping studies offers several advantages, making it a popular choice for genetic research and diversity assessment, providing excellent separation of DNA fragments based on their size and allowing for precise determination of allele sizes. This high resolution is crucial for distinguishing different alleles in microsatellite regions, allowing for accurate differentiation between homozygous and heterozygous individuals, providing detailed genotype information. CE with SSR markers is widely used in various genetic research areas, such as population genetics, phylogenetics, and evolutionary studies [27].

In the present study, the combination of highly polymorphic SSR markers and fragment analysis by CE was implemented for the molecular characterization and assessment of the genetic diversity of local walnut trees that were collected in the Parnon mountain range in Peloponnese. In summary, the selected SSR markers proved to be very informative about the high genetic variation of the collected walnut genotypes, although low genetic distances between the individuals were found. Also, no correlation was found between the observed genetic variability and the collection sites of the walnut samples.

## 2. Results

### 2.1. Polymorphism of SSR Primer Pairs

Nine SSR primer pairs were employed to assess the genetic diversity and population structure of 47 walnut genotypes. Across these 47 walnut genotypes, a total of 242 polymorphic bands were identified for all the markers used. The number of alleles per primer pair ranged from 19 to 42 (as detailed in Supplementary Table S1).

**Figure 1:**
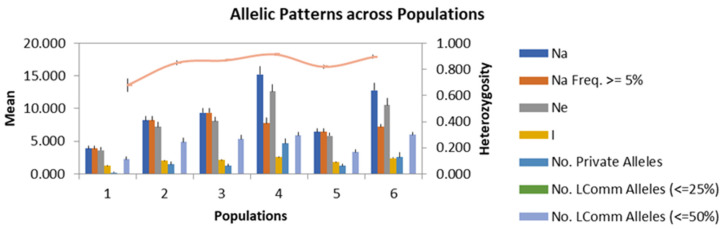
Allelic patterns for all SSR markers across the six groups that formed according to the altitude range.

In our analysis, the values for Na ranged from 6.5 to 12.83, with an average of 9.3. Similarly, Ne values spanned from 5.94 to 11.27, with a mean of 7.97. As for He values, they varied from 0.76 to 0.88, with an average of 0.84. In contrast, Ho values ranged between 0.00 and 0.84, with an average of 0.47. Shannon’s Information Index exhibited a range from 1.70 to 2.37, with an average of 2.03. Allelic patterns across the six groups of walnut samples are depicted in Figure 1.

Moreover, the PIC values for each primer pair fell within the range of 0.93 to 0.97, with an average of 0.94, and the PI values ranged from 0.0019 to 0.0102, with an average of 0.0061 (as summarized in Table 1).

### 2.2. Population Structure Analysis

**Figure 2:**
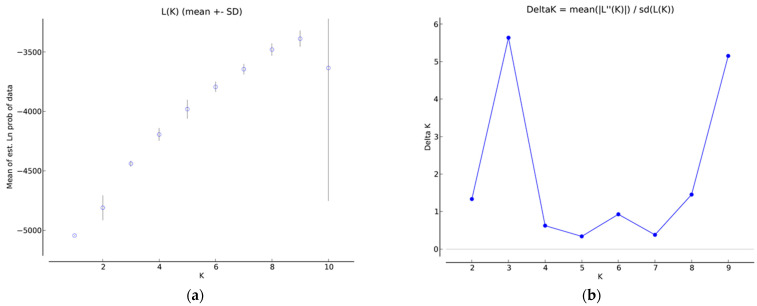
(**a**) LnP(D) and (**b**) ΔK evaluation of the 47 walnut genotypes, displaying a distinct peak at the value of K = 3.

The genetic structure of the 47 walnut genotypes was inferred using SSR markers. The LnP(D) score, which estimates the posterior probability of the data for a specific K (the number of populations), increased as K increased, and ΔK reached its highest point at K = 3. Essentially, the STRUCTURE program, when utilizing the admixture model with correlated allele frequencies, and the ad hoc metric based on the second-order rate of change of the likelihood function (ΔK) [30] displayed a distinct peak at the true value of K = 3 (as shown in Figure 2).

**Figure 3:**
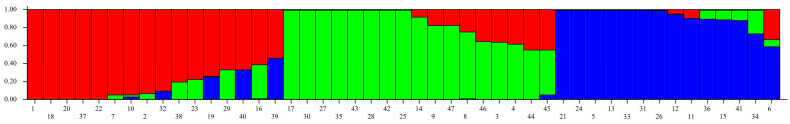
Structure of the 47 walnut genotypes based on the analysis of nine SSR markers (K = 3). Each color (red, green and blue) corresponds to each cluster of samples.

Consequently, the 47 genotypes were categorized into three clusters, irrespective of their geographic origin or altitude level. The first cluster consisted of individuals 05, 06, 24, 36, 34, 15, 21, 26, 31, 33, 13, 12, 41, and 11, with altitudes ranging from 720 to 1297 m. The average genetic distance (expected heterozygosity) between individuals in this cluster was 0.9087. The second cluster included genotypes 07, 40, 22, 45, 30, 42, 28, 17, 35, 47, 43, 04, 46, 44, 08, 03, 27, 14, and 09, with altitudes between 730 and 1224 m. The average genetic distance (expected heterozygosity) among these individuals was 0.9186. Finally, the third cluster contained individuals with altitudes ranging from 840 to 1290 m, such as genotypes 25, 23, 18, 16, 01, 02, 39, 37, 20, 19, 32, 10, 29, and 38. The average genetic distance (expected heterozygosity) within this cluster was 0.8940 (depicted in Figure 3).

As most of the walnut accessions examined in this study were mainly obtained from the Parnon mountain region, the findings of the population structure analysis were applied onto a map. The outcomes indicated that the observed grouping was not influenced by their geographical source or altitude level.

### 2.3. Principal Coordinate Analysis

**Figure 4:**
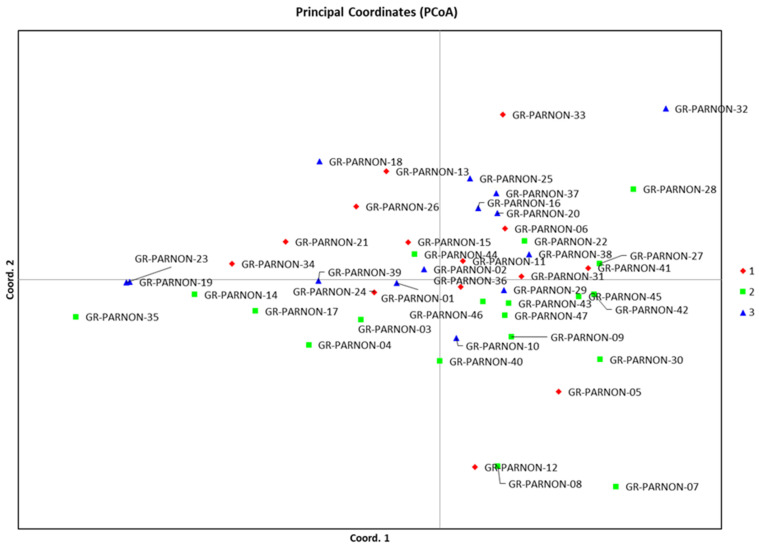
Principal Coordinate Analysis (PCoA) diagram of the 47 walnut genotypes, showing a clear overlap among the three clusters, a result of low genetic distances among the studied individuals.

Principal Coordinate Analysis (PCoA) was conducted to validate the outcomes of the population structure analysis. The three principal coordinates accounted for 6.96%, 6.61%, and 6.39% of the molecular variance, summing up to a total of 19.95% (as shown in Figure 4). All the examined genotypes were categorized in accordance with the results of the population structure analysis. The PCoA revealed that there was a considerable overlap among all three clusters, and the genetic distance separating them was relatively small. However, specific individuals, such as GR-Parnon 32, GR-Parnon 33, and GR-Parnon 07, were observed to exhibit differentiation from the main clusters (as depicted in Figure 4).

### 2.4. UPGMA Cluster Analysis

**Figure 5:**
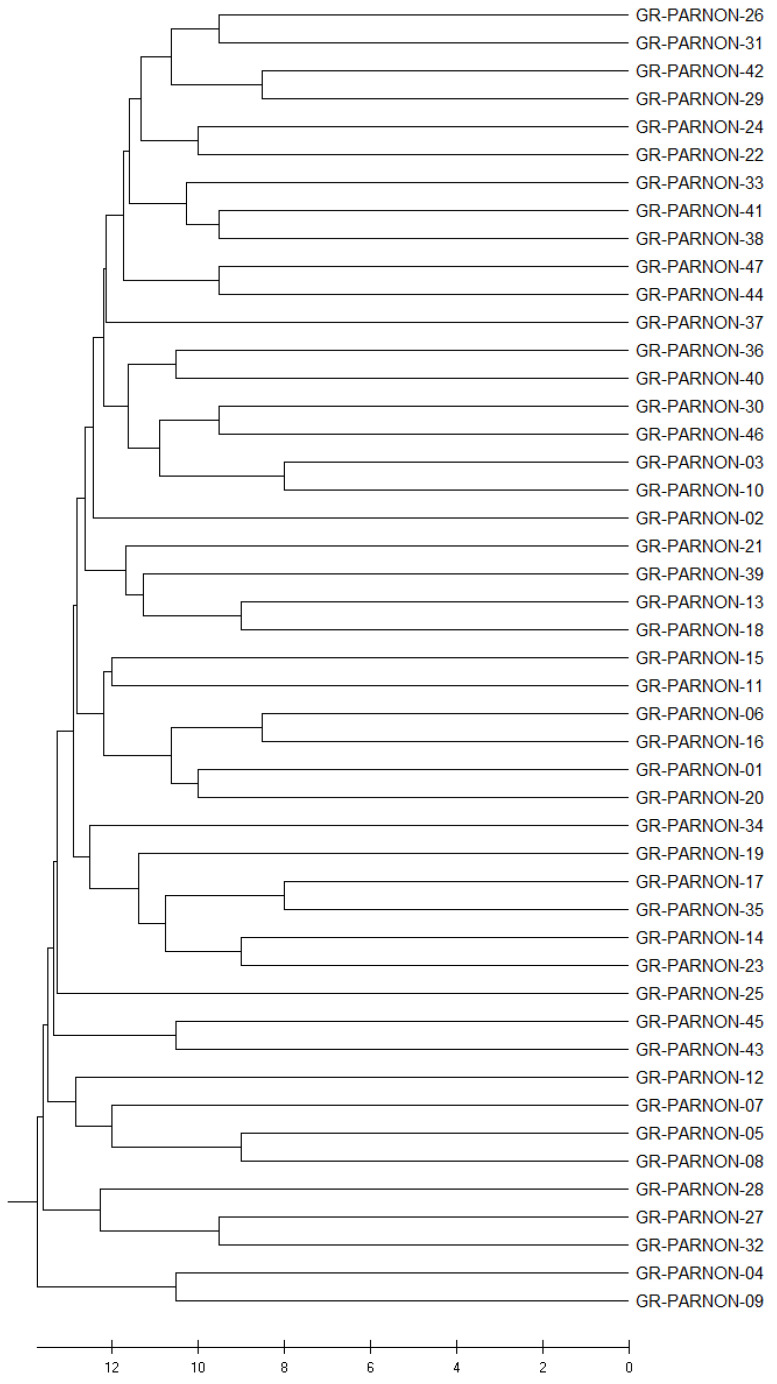
The dendrogram of the 47 walnut genotypes was inferred using the UPGMA method, where small different subgroups of individuals were formed. The optimal tree with a branch length sum of 522.58917747 is shown. The tree is drawn to scale, with branch lengths in the same units as those of the genetic distances used to infer the dendrogram.

The UPGMA cluster analysis showed that the walnut genotypes from the same origin did not exhibit obvious clustering, and small different groups of individuals were formed, as seen in Figure 5.

### 2.5. Genetic Diversity Analysis

The genetic differentiations of the 47 walnut genotypes were analyzed using AMOVA. The results of AMOVA for the SSR markers revealed a percentage of 100% within populations. The results from Shannon’s Information Diversity Statistics indicated that 27% of the total diversity was attributed to the diversity among clusters and 73% was attributed to the diversity within clusters.

## 3. Discussion

Walnut trees exhibit a wide-ranging genetic diversity, with significant differentiation present within walnut germplasm [31]. The observed genetic variation and the heterozygosity among the walnut genotypes primarily originates from plantations grown from seeds [32]. Evaluating the available germplasm is essential for future crop improvement programs [33,34], and the examination of these resources for diverse characteristics yields valuable insights for the selection of advantageous genetic assets [35,36].

Molecular markers offer a rich source of data about genome structure and evolution [37]. They prove invaluable for tasks such as mapping both qualitative and quantitative traits, constructing linkage maps [38], improving germplasm, conducting genotyping, identifying parentage, and exploring population genetics [39]. Furthermore, through genome-wide association studies, it becomes feasible to identify and pinpoint genes associated with crucial traits, facilitating marker-assisted selection (MAS) and gene cloning [40].

In our study, we estimated the genetic diversity of 47 walnut trees through the utilization of SSR markers initially developed in *J. nigra* [41]. The results acquired indicate that the chosen SSR markers effectively enable the differentiation of genetic diversity among the walnut tree genotypes originating from the north-eastern Parnon mountain region. Notably, all the primers employed in this study demonstrated a 100% polymorphism rate, a finding congruent with the outcomes reported by Ahmed et al. [14] and Kabiri et al. [17]. This discovery aligns with numerous other studies that have also employed these markers to explore genetic diversity in walnut trees, such as those conducted by Ruiz-Garcia et al. [42], Ebrahimi et al. [43], and Vischi et al. [44].

The highest average number of alleles per locus, which amounted to 23.8, was documented by Victory et al. [45], while Zhang et al. [46] observed the lowest number at 3. Meanwhile, other researchers have reported values ranging from 4.25 to 12 alleles per locus in their studies, such as Itoo et al. [47], Orhan et al. [48], Balapanov et al. [20], Vahdati et al. [49], Mahmoodi et al. [50], Ebrahimi et al. [51], and Dangl et al. [21].

In our current investigation, we identified a total of 242 alleles, with an average of 9.3 alleles per locus in the genetic profiling of 47 genotypes. These findings align with previous studies conducted by Balapanov et al. [20], where they also reported relatively high average numbers of alleles—specifically, 9.6 and 9.4.

Variations in the number of alleles, whether lower or higher, can be attributed to several factors, including the composition of the study group in terms of germplasm diversity, the types of markers used, the geographical characteristics of the sampled region, and other relevant factors [19,52]. It is worth noting that minor disparities in polymorphic alleles across different studies may arise from the specific choice of genotypes or cultivars. Nevertheless, the outcomes of our study are consistent with prior investigations into genetic diversity among walnut genotypes using SSR markers. It is important to acknowledge that the genetic and phylogenetic distances among the genotypes can also influence the level of polymorphism detected by SSR markers, particularly owing to the outcrossing nature of walnuts [26].

In recent investigations, the observed heterozygosity has been reported within the range of 0.57 to 1.00 (with an average of 0.80) as noted by Eser et al. [53], 0.633 to 0.895 (with an average of 0.75) according to Balapanov et al. [20], 0.548 to 0.927 (with an average of 0.803) in the research conducted by Zhou et al. [54], 0.39 to 0.80 (with an average of 0.60) in a study by Orhan et al. [48], and 0.250 to 0.833 (with an average of 0.514) as documented by Bujdoso and Cseke [55]. In this study, the average Ho ranged from 0.00 to 0.84, with a mean of 0.47, which agrees with the Ho of around 0.5 reported by Aradhya et al. [56] for the region of Eurasia, while for walnut populations from Central Asia, Western Asia, and the Middle East, Pollegioni et al. [57] found 0.559.

Furthermore, in our study, we found that the expected heterozygosity (He = 0.84) exceeded the observed heterozygosity (Ho = 0.47), a pattern that has been previously documented in various research endeavors [20,34,42,48,50,58,59]. This discrepancy suggests a potential deficiency of heterozygotes within the genotypes or populations, which could be attributed to factors such as unrestricted inbreeding, selective mating, a small sample population size, and other related variables.

The PIC values, which consider the number of alleles per locus and their relative frequencies in the population, serve as indicators of the discriminatory potential of a given locus. Markers with PIC values greater than 0.5 are considered informative, while those exceeding 0.7 are deemed well-suited for genetic mapping. Both types of markers can significantly enrich our understanding of walnut breeding and genetics [48]. Furthermore, an analysis of the Probability of Identity (PI), which is implemented as an individual identification estimator, showed very low values for each SSR marker, with an average of 0.0061, which is within the range reported by Waits and colleagues (2001) [60].

Recent research studies have reported a range of PIC values from 0.15 to 0.86. For instance, Guney et al. [58] found PIC values falling within the range of 0.42 to 0.86, with an average of 0.68, while Orhan et al. [48] observed PIC values ranging from 0.54 to 0.85, with an average of 0.68. In a study conducted by Bernard et al. [61], the PIC values ranged from 0.15 to 0.75, with an average of 0.52, and Vahdati et al. [49] reported PIC values spanning from 0.56 to 0.82, with an average of 0.72. These variations in PIC values across different research studies can be attributed to factors such as the use of different types and quantities of SSR markers and variations in the number and sampling locations of specimens [48].

It is noteworthy that all SSR markers used in this study demonstrated high PIC values, indicating that the chosen markers were well-suited for evaluating the genetic diversity among the 47 walnut genotypes. Also, we observed that the average PIC value was higher when compared to the previously mentioned studies. These differences in PIC values among studies may arise from the utilization of distinct SSR markers or from the origins of samples, which could encompass considerably more diverse regions, potentially spanning different continents [26]. Notably, the Eurasia region, which includes Greece, has been recognized for its notably high levels of genetic diversity in walnuts [57].

The results from STRUCTURE indicated the formation of three different clusters irrespective of the geographic origin or altitude level of the collected walnut tree samples. Furthermore, PCoA revealed that there was extensive overlap among the walnut genotypes belonging to all three clusters, and the genetic distances separating most of them were relatively small, but a considerable number of individuals exhibited higher genetic distances. These findings can be explained by the presence of old walnut tree plantations, mainly seed-based plantations, in the selected area of the Parnon mountain range. These individuals could prove valuable resources of important genetic traits for future breeding programs. The UPGMA dendrogram validated the PCoA findings, where many subclusters were formed.

## 4. Materials and Methods

### 4.1. Plant Material

**Figure 6:**
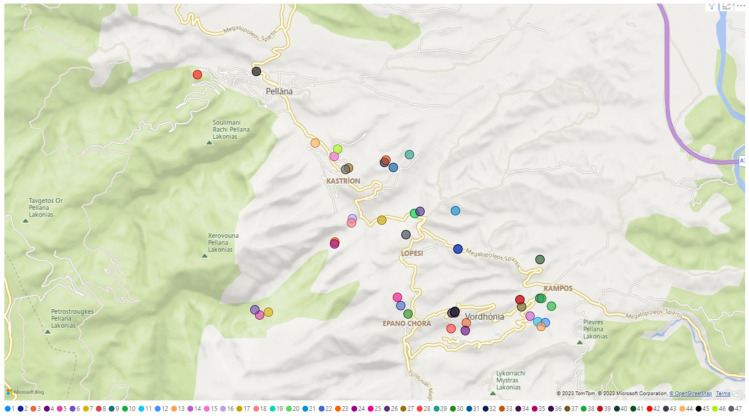
Map of the north-east Parnon mountain region, where the 47 *J. regia* genotypes were collected (generated by PowerBI).

Fresh leaves from 47 walnut tree samples were collected from the north-east Parnon mountain region (Table 2 and Figure 6) and stored at −20 °C. 

### 4.2. Molecular Analysis Using SSR Markers

DNA extraction from fresh leaves was performed according to the CTAB (cetyl trime-thylammonium bromide) protocol as described by Doyle and Doyle [62]. DNA quality evaluation and quantification was performed by using a Quawell UV–Vis Spectrophotometer Q5000 (Quawell Technology, Inc., San Jose, CA, USA). Nine SSR molecular markers were selected after a systematic review of the available scientific literature for the genotypic characterization of walnut cultivars, and they were developed by Woeste et al. [41]. Information on the SSR primer pairs is shown in Table 2. PCR amplifications for the SSR markers were performed in 25 μL reaction volumes containing 20 ng of genomic DNA, 1× PCR buffer, 0.5 μM of primer, 0.2 mM of DNTPs, and 1 U of Kapa Taq polymerase. PCR reactions for each SSR marker were performed using a SureCycler 8800 thermocycler (Agilent Technologies, Santa Clara, CA, USA) under the following thermal cycling conditions: a first step at 94 °C for 5 min; followed by 35 cycles segmented in 30 s at 94 °C, 30 s at varied annealing temperatures based on the primers (Table 3), and 30 s at 72 °C; and a final extension at 72 °C for 5 min. The PCR products were analyzed on a 2% agarose gel in 1× TAE buffer. The Quick-Load^®^ 1 kb Plus DNA Ladder (New England Biolabs, Ipswich, MA, USA) was used as a molecular weight size marker in each gel, in order to validate the expected size of the PCR products. Further analysis of the PCR products was performed by means of fragment analysis using the dsDNA 915 Reagent kit (Agilent, Santa Clara, CA, USA) in the 5200 Fragment Analyzer System (Agilent, Santa Clara, CA, USA). The dsDNA 915 Reagent kit is suitable for the analysis of dsDNA fragments between 35 and 5000 bp. This specific genetic analysis method comprises a series of techniques in which DNA fragments are fluorescently labeled, separated by capillary electrophoresis (CE), and sized by comparison to an internal standard. Also, relative quantification between samples can be obtained using this kit.

### 4.3. Molecular Data Analysis

The size of each amplified allele was determined by comparison with an internal standard (Ladder 35–5000 bp) in the ProSize v3.0 data analysis software (Agilent, Santa Clara, CA, USA). Allele frequencies were computed, and the distance matrix derived from the nine SSR markers was employed for several analyses. For the statistical analysis of the molecular data, the 47 walnut genotypes were clustered into six groups according to their collection site altitudes (Table 4). The analysis comprised a calculation of the polymorphic information content (PIC) for each primer, to assess the markers’ utility and effectiveness, and also the Probability of Identity (PI) for each marker, through Cervus 3.0.7 software [63]. Additionally, various parameters, including the number of alleles (Na), effective number of alleles (Ne), expected heterozygosity (He), observed heterozygosity (Ho), and Shannon’s Index (I), were determined. Furthermore, we computed the variation in genetic diversity (Shannon Informational Diversity) in relation to altitude levels. Further genetic analyses involved an Analysis of Molecular Variance (AMOVA) and Principal Coordinate Analysis (PCoA). All these analyses were performed using GenAlEx 6.5 [64,65] as the analytical tool. The genetic distance matrix was applied to construct the Unweighted Pair Group Method with Arithmetic mean (UPGMA) tree [66], facilitated by MEGA X [67].

To enhance the clustering of walnut genotypes, we employed the model-based Bayesian clustering algorithm within STRUCTURE v.2.3.4 [68]. For each K value (ranging from 1 to 10), STRUCTURE was independently executed 10 times, with a burn-in period of 10,000 iterations and 100,000 iterations for MCMC (Markov chain Monte Carlo). The analysis utilized the admixture model with correlated allele frequencies, as proposed by Falush et al. [69]. To determine the optimal number K of clusters that best explained the observed genetic structure, we employed the STRUCTURE Harvester website [70] and implemented the Evanno method [30].

## 5. Conclusions

The fundamental objective of any diversity assessment is to quantify the extent of variation within a population, pinpoint and choose outstanding plants, and preserve these seedling trees for future utilization. In summary, SSR markers are proven to be a valuable tool for assessing genetic diversity and identifying genetically unique or rare walnut genotypes, due to their high polymorphism, co-dominant inheritance, and ease of use. By understanding the genetic diversity present in walnut tree populations, breeders can make informed decisions about which trees to cross in order to develop new walnut varieties with improved characteristics, such as disease resistance, nut size, and taste.

In our study, high levels of genetic variation were found among the individual walnut genotypes that were collected in the Parnon mountain region. Our results also suggest that there is no significant correlation between the observed genetic variability and the geographic origin or altitude of the collected walnut genotypes. This fact contributed to the formation of overlapping clusters. Our findings suggest that the collected walnut tree genotypes belong to one population—that of the geographic origin of the Parnon mountain range—and are characterized by high levels of genetic diversity. The genetic diversity of Greek genotypes could substantially contribute towards the development of new varieties in walnut breeding efforts. Thus, Greece could be considered a long-established region of walnut diversity with a vast gene pool resource. The genetic diversity of traditional Greek walnut tree populations is being rapidly lost due to the introduction of international cultivars. Thus, the conservation of Greek walnut tree landrace genotypes is very important.

Moreover, the variation in agronomic traits of Greek walnut tree populations would be of great interest for future studies and future cultivar improvement. Future studies on Greek *J. regia* genotypes are expected to employ broader and more comprehensive samplings across different regions in Greece. These studies will incorporate genetic analysis with microsatellite markers, along with correlation of the SSR markers and phenotypic traits. This approach will contribute to a more detailed and comprehensive exploration of the Greek walnut population’s genetic architecture.

## Figures and Tables

**Table 1:** Number of different alleles (Na), number of effective alleles (Ne), observed heterozygosity (Ho), expected heterozygosity (He), Shannon’s Information Index (I), polymorphism information content (PIC), and Probability of Identity (PI) from 47 walnut genotypes.

Primer	Na	Ne	Ho	He	I	PIC	PI
WGA009	9.50	7.78	0.54	0.84	2.03	0.94	0.0073
WGA321	9.50	8.07	0.62	0.85	2.06	0.95	0.0059
WGA349	9.17	7.50	0.57	0.84	2.04	0.94	0.0071
WGA069	9.83	8.46	0.60	0.87	2.17	0.95	0.0057
WGA118	6.50	5.94	0.03	0.76	1.70	0.93	0.0102
WGA202	6.67	6.20	0.00	0.78	1.73	0.94	0.0059
WGA276	9.17	7.33	0.54	0.83	2.00	0.94	0.0073
WGA001	12.83	11.27	0.84	0.88	2.37	0.97	0.0019
WGA376	10.67	9.23	0.53	0.87	2.20	0.96	0.0035
Mean	9.3	7.97	0.47	0.84	2.03	0.94	0.0061

**Table 2:** Coordinates where the local walnut genotypes were collected in the north-east region of the Parnon mountain range, in Greece.

No.	Local Walnut Selection	Latitude	Longitude	Altitude
1	GR-PARNON-01	37°15′13.8″ N	22°33′14.2″ E	1031
2	GR-PARNON-02	37°15′13.3″ N	22°33′14.2″ E	1011
3	GR-PARNON-03	37°14′27.1″ N	22°33′26.5″ E	1079
4	GR-PARNON-04	37°14′18.1″ N	22°33′25.0″ E	1045
5	GR-PARNON-05	37°14′36.3″ N	22°30′22.4″ E	720
6	GR-PARNON-06	37°14′42.4″ N	22°30′15.5″ E	755
7	GR-PARNON-07	37°14′39.6″ N	22°30′35.4″ E	730
8	GR-PARNON-08	37°14′54.1″ N	22°34′05.3″ E	1062
9	GR-PARNON-09	37°14′55.8″ N	22°34′34.6″ E	1224
10	GR-PARNON-10	37°14′55.7″ N	22°34′37.3″ E	1247
11	GR-PARNON-11	37°14′28.5″ N	22°34′31.6″ E	1297
12	GR-PARNON-12	37°14′27.3″ N	22°34′42.7″ E	1280
13	GR-PARNON-13	37°14′22.9″ N	22°34′36.6″ E	1278
14	GR-PARNON-14	37°14′35.0″ N	22°34′20.3″ E	1212
15	GR-PARNON-15	37°16′22.1″ N	22°31′32.1″ E	1020
16	GR-PARNON-16	37°15′49.1″ N	22°31′58.9″ E	950
17	GR-PARNON-17	37°15′47.4″ N	22°32′02.3″ E	980
18	GR-PARNON-18	37°15′44.2″ N	22°31′57.6″ E	940
19	GR-PARNON-19	37°15′55.4″ N	22°32′50.1″ E	1135
20	GR-PARNON-20	37°15′55.0″ N	22°32′51.0″ E	1105
21	GR-PARNON-21	37°15′58.4″ N	22°33′10.5″ E	1100
22	GR-PARNON-22	37°14′47.2″ N	22°32′30.1″ E	865
23	GR-PARNON-23	37°15′21.7″ N	22°31′33.2″ E	910
24	GR-PARNON-24	37°15′19.5″ N	22°31′32.7″ E	878
25	GR-PARNON-25	37°14′57.0″ N	22°32′25.2″ E	840
26	GR-PARNON-26	37°15′57.6″ N	22°32′58.4″ E	1127
27	GR-PARNON-27	37°16′08.6″ N	22°31′53.3″ E	1110
28	GR-PARNON-28	37°14′20.3″ N	22°33′04.1″ E	921
29	GR-PARNON-29	37°16′24.3″ N	22°32′42.9″ E	1279
30	GR-PARNON-30	37°14′37.5″ N	22°32′40.8″ E	884
31	GR-PARNON-31	37°16′09.3″ N	22°32′19.1″ E	1204
32	GR-PARNON-32	37°16′15.1″ N	22°32′06.1″ E	1217
33	GR-PARNON-33	37°16′17.8″ N	22°32′08.3″ E	1232
34	GR-PARNON-34	37°14′40.6″ N	22°33′09.9″ E	985
35	GR-PARNON-35	37°14′38.7″ N	22°33′09.5″ E	1001
36	GR-PARNON-36	37°14′38.6″ N	22°33′05.4″ E	950
37	GR-PARNON-37	37°14′45.9″ N	22°34′07.8″ E	1078
38	GR-PARNON-38	37°14′46.6″ N	22°34′51.7″ E	1290
39	GR-PARNON-39	37°14′54.1″ N	22°34′05.4″ E	1061
40	GR-PARNON-40	37°14′39.7″ N	22°33′09.5″ E	803
41	GR-PARNON-41	37°15′01.2″ N	22°34′34.9″ E	1287
42	GR-PARNON-42	37°17′18.0″ N	22°29′31.0″ E	905
43	GR-PARNON-43	37°15′30.4″ N	22°32′37.7″ E	1041
44	GR-PARNON-44	37°16′37.9″ N	22°31′04.2″ E	1053
45	GR-PARNON-45	37°17′21.8″ N	22°30′17.7″ E	880
46	GR-PARNON-46	37°16′30.7″ N	22°31′37.3″ E	1052
47	GR-PARNON-47	37°16′06.8″ N	22°31′48.9″ E	1036

**Table 3:** Details and characteristics of 9 SSR primers used across 47 genotypes of walnut.

Primer	Primer Sequence (5′–3′)	Repeat Motif	Annealing Temperature (°C)	Product Size Range (bp)
WGA009	F CATCAAAGCAAGCAATGGG	(GA)_16_	51	231–245
	R CCATTGCTCTGTGATTGGG			
WGA321	F TCCAATCGAAACTCCAAAGG	(GA)_14_	51	223–245
	R TGTCCAAAGACGATGATGGA			
WGA349	F GTGGCGAAAGTTTATTTTTTGC	(CT)_14_	52	262–274
	R ACAAATGCACAGCAGCAAAC			
WGA069	F TTAGTTAGCAAACCCACCCG	(GA)_4_ATATAA(GA)_1_	54	160–182
	R AGATGCACAGACCAACCCTC			
WGA118	F TGTGCTCTGATCTGCCTCC	(GA)_18_(GT)_11_	54	186–200
	R GGGTGGGTGAAAAGTAGCAA			
WGA202	F CCCATCTACCGTTGCACTTT	(GA)_11_	54	259–295
	R GCTGGTGGTTCTATCATGGG			
WGA276	F CTCACTTTCTCGGCTCTTCC	(GA)_14_	55	168–194
	R GGTCTTATGTGGGCAGTCGT			
WGA001	F ATTGGAAGGGAAGGGAAATG	(GA)_5_GCA(GA)_3_GCA(GA)_3_	52	180–192
	R CGCGCACATACGTAAATCAC			
WGA376	F GCCCTCAAAGTGATGAACGT	(AG)_2_AA(AG)_6_	54	230–265
	R TCATCCATATTTACCCCTTTCG			

**Table 4:** The 47 walnut samples were grouped together for the marker analysis according to their altitude range.

Groups of Samples	Altitude Range
1	720–755 m
2	803–884 m
3	905–985 m
4	1001–1079 m
5	1100–1135 m
6	1204–1297 m

## Data Availability

The data presented in this study are available on request from the corresponding author.

## Supplementary Materials

The following supporting information can be downloaded at: https://www.mdpi.com/article/10.3390/ijms242417230/s1.

## References

[1] FAOSTAT. https://www.fao.org/faostat/en/.

[2] Halvorsen B.L., Carlsen M.H., Phillips K.M., Bøhn S.K., Holte K., Jacobs D.R., Blomhoff R. (2006). Content of Redox-Active Compounds (Ie, Antioxidants) in Foods Consumed in the United States. Am. J. Clin. Nutr..

[3] Kornsteiner M., Wagner K.-H., Elmadfa I. (2006). Tocopherols and Total Phenolics in 10 Different Nut Types. Food Chem..

[4] Hayes D., Angove M.J., Tucci J., Dennis C. (2016). Walnuts (*Juglans regia*) Chemical Composition and Research in Human Health. Crit. Rev. Food Sci. Nutr..

[5] Ros E., Izquierdo-Pulido M., Sala-Vila A. (2018). Beneficial Effects of Walnut Consumption on Human Health: Role of Micronutrients. Curr. Opin. Clin. Nutr. Metab. Care.

[6] Liu X., Guasch-Ferré M., Tobias D.K., Li Y. (2021). Association of Walnut Consumption with Total and Cause-Specific Mortality and Life Expectancy in U.S. Adults. Nutrients.

[7] Germain E., Aletà N., Ninot A., Rouskas D., Zakinthinos G., Pereira G., Monastra F. (1997). Prospections réalisées dansles populations de semis de noyer d’Espagne, de Grèce, d’Italie etdu Portugal: Caractérisation des populations et description en collections d’études des présélections issues deces prospections. Options Méditerr.

[8] Rouskas D., Katranis N., Zakynthinos G., Isaakidis R. (1997). Walnuts (*Junglans regia* L.) Seedlings Selection in Greece. Acta Hortic..

[9] Rouskas D., Zakynthinos G. (2001). Preliminary Evaluation of Seventy Walnut (*Juglans regia* L.) Seedlings Selections in Greece. Acta Hortic..

[10] Christopoulos M.V., Rouskas D., Tsantili E., Bebeli P.J. (2010). Germplasm Diversity and Genetic Relationships among Walnut (*Juglans regia* L.) Cultivars and Greek Local Selections Revealed by Inter-Simple Sequence Repeat (ISSR) Markers. Sci. Hortic..

[11] Avanzato D. (2014). Following Walnut Footprints (Juglans regia L.): Cultivation and Culture, Folklore and History, Traditions and Uses.

[12] Hellenic Statistical Authority–ELSTAT. https://www.statistics.gr/el/statistics/.

[13] Bayazit S., Kazan K., Gülbitti S., Çevik V., Ayanoğlu H., Ergül A. (2007). AFLP Analysis of Genetic Diversity in Low Chill Requiring Walnut (*Juglans regia* L.) Genotypes from Hatay, Turkey. Sci. Hortic..

[14] Ahmed N., Mir J.I., Mir R.R., Rather N.A., Rashid R., Wani S.H., Shafi W., Mir H., Sheikh M.A. (2012). SSR and RAPD Analysis of Genetic Diversity in Walnut (*Juglans regia* L.) Genotypes from Jammu and Kashmir, India. Physiol. Mol. Biol. Plants.

[15] Nickravesh M.H., Vahdati K., Amini F., Amiri R., Woeste K. (2021). Application of Microsatellite and Inter Simple Sequence Markers for Certifying Trueness to Type of Grafted Ramets and Clustering of Persian Walnut Varieties. Res. Sq..

[16] Ji A., Wang Y., Wu G., Wu W., Yang H., Wang Q. (2014). Genetic Diversity and Population Structure of North China Mountain Walnut Revealed by ISSR. Am. J. Plant Sci..

[17] Kabiri G., Bouda S., Haddioui A. (2022). Evaluation of Genetic Diversity and Structuration across Altitude of Walnut (*Juglans regia* L.) Accessions from Morocco Using SSR Markers. Bulg. J. Agric. Sci..

[18] Bernard A., Marrano A., Donkpegan A., Brown P.J., Leslie C.A., Neale D.B., Lheureux F., Dirlewanger E. (2020). Association and Linkage Mapping to Unravel Genetic Architecture of Phenological Traits and Lateral Bearing in Persian Walnut (*Juglans regia* L.). BMC Genom..

[19] Shah R.A., Baksi P., Jasrotia A., Bhat D.J., Gupta R., Bakshi M. (2020). Genetic Diversity of Walnut (*Juglans regia* L.) Seedlings through SSR Markers in North-Western Himalayan Region of Jammu. Bangladesh J. Bot..

[20] Balapanov I., Suprun I., Stepanov I., Tokmakov S., Lugovskoy A. (2019). Comparative Analysis Crimean, Moldavian and Kuban Persian Walnut Collections Genetic Variability by SSR-Markers. Sci. Hortic..

[21] Dangl G.S., Woeste K., Aradhya M.K., Koehmstedt A., Simon C., Potter D., Leslie C.A., McGranahan G. (2005). Characterization of 14 Microsatellite Markers for Genetic Analysis and Cultivar Identification of Walnut. J. Am. Soc. Hortic. Sci..

[22] Mohsenipoor S., Vahdati K., Amiri R., Mozaffari M.R. (2010). Study of the Genetic Structure and Gene Flow in Persian Walnut (*Juglans regia* L.) Using SSR Markers. Acta Hortic..

[23] Bükücü Ş.B., Sütyemez M., Kefayati S., Paizila A., Jighly A., Kafkas S. (2020). Major QTL with Pleiotropic Effects Controlling Time of Leaf Budburst and Flowering-Related Traits in Walnut (*Juglans regia* L.). Sci. Rep..

[24] Aradhya M.K., Velasco D., Wang J., Ramasamy R., You F.M., Leslie C., Dandekar A., Luo M., Dvorak J. (2019). A Fine-scale Genetic Linkage Map Reveals Genomic Regions Associated with Economic Traits in Walnut (*Juglans regia*). Plant Breed..

[25] Foroni I., Rao R., Woeste K., Gallitelli M. (2005). Characterisation of *Juglans regia* L. with SSR Markers and Evaluation of Genetic Relationships among Cultivars and the ‘Sorrento’ Landrace. J. Hortic. Sci. Biotechnol..

[26] Shah R.A., Bakshi P., Jasrotia A., Itoo H., Padder B.A., Gupta R., Kour G., Dolkar D. (2023). Morphological to Molecular Markers: Plant Genetic Diversity Studies in Walnut (*Juglans regia* L.)—A Review. Erwerbs-Obstbau.

[27] Guichoux E., Lagache L., Wagner S., Chaumeil P., Léger P., Lepais O., Lepoittevin C., Malausa T., Revardel E., Salin F. (2011). Current Trends in Microsatellite Genotyping. Mol. Ecol. Resour..

[28] Boopathi N.M. (2020). Genotyping of Mapping Population. Genetic Mapping and Marker Assisted Selection.

[29] Szantai E., Ronai Z., Szilagyi A., Sasvari-Szekely M., Guttman A. (2005). Haplotyping by Capillary Electrophoresis. J. Chromatogr. A.

[30] Evanno G., Regnaut S., Goudet J. (2005). Detecting the Number of Clusters of Individuals Using the Software structure: A Simulation Study. Mol. Ecol..

[31] Shahi Shavvon R., Qi H.-L., Mafakheri M., Fan P.-Z., Wu H.-Y., Bazdid Vahdati F., Al-Shmgani H.S., Wang Y.-H., Liu J. (2023). Unravelling the Genetic Diversity and Population Structure of Common Walnut in the Iranian Plateau. BMC Plant Biol..

[32] Büyüksolak Z.N., Aşkın M.A., Kahramanoğlu İ., Okatan V. (2020). Effects of Altitude on the Pomological Characteristics and Chemical Properties of ‘Chandler’ Walnuts: A Case Study in Uşak Province. Acta Agrobot..

[33] Akça Y., Yuldaşulu Y.B., Murad E., Vahdati K. (2020). Exploring of Walnut Genetic Resources in Kazakhstan and Evaluation of Promising Selections. Int. J. Hortic. Sci. Technol..

[34] Pop I.F., Vicol A.C., Botu M., Raica P.A., Vahdati K., Pamfil D. (2013). Relationships of Walnut Cultivars in a Germplasm Collection: Comparative Analysis of Phenotypic and Molecular Data. Sci. Hortic..

[35] Ebrahimi A., Khadivi-Khub A., Nosrati Z., Karimi R. (2015). Identification of Superior Walnut (*Juglans regia*) Genotypes with Late Leafing and High Kernel Quality in Iran. Sci. Hortic..

[36] Hassani D., Sarikhani S., Dastjerdi R., Mahmoudi R., Soleimani A., Vahdati K. (2020). Situation and Recent Trends on Cultivation and Breeding of Persian Walnut in Iran. Sci. Hortic..

[37] Cervera M.T., Plomion C., Malpica C., Jain S.M., Minocha S.C. (2000). Molecular Markers and Genome Mapping in Woody Plants. Molecular Biology of Woody Plants.

[38] Crespel L., Chirollet M., Durel C., Zhang D., Meynet J., Gudin S. (2002). Mapping of Qualitative and Quantitative Phenotypic Traits in Rosa Using AFLP Markers. Theor. Appl. Genet..

[39] Priyanka V., Kumar R., Dhaliwal I., Kaushik P. (2021). Germplasm Conservation: Instrumental in Agricultural Biodiversity—A Review. Sustainability.

[40] De La Rosa R., Angiolillo A., Guerrero C., Pellegrini M., Rallo L., Besnard G., Bervillé A., Martin A., Baldoni L. (2003). A First Linkage Map of Olive (*Olea europaea* L.) Cultivars Using RAPD, AFLP, RFLP and SSR Markers. Theor. Appl. Genet..

[41] Woeste K. (2002). Thirty Polymorphic Nuclear Microsatellite Loci from Black Walnut. J. Hered..

[42] Ruiz-Garcia L., Lopez-Ortega G., Fuentes Denia A., Frutos Tomas D. (2011). Identification of a Walnut (*Juglans regia* L.) Germplasm Collection and Evaluation of Their Genetic Variability by Microsatellite Markers. Span. J. Agric. Res..

[43] Ebrahimi A., Zarei A., Lawson S., Woeste K.E., Smulders M.J.M. (2016). Genetic Diversity and Genetic Structure of Persian Walnut (*Juglans regia*) Accessions from 14 European, African, and Asian Countries Using SSR Markers. Tree Genet. Genomes.

[44] Vischi M., Chiabà C., Raranciuc S., Poggetti L., Messina R., Ermacora P., Cipriani G., Paffetti D., Vettori C., Testolin R. (2017). Genetic Diversity of Walnut (*Juglans regia* L.) in the Eastern Italian Alps. Forests.

[45] Victory E.R., Glaubitz J.C., Rhodes O.E., Woeste K.E. (2006). Genetic Homogeneity in Juglans Nigra (Juglandaceae) at Nuclear Microsatellites. Am. J. Bot..

[46] Zhang R., Zhu A., Wang X., Yu J., Zhang H., Gao J., Cheng Y., Deng X. (2010). Development of *Juglans regia* SSR Markers by Data Mining of the EST Database. Plant Mol. Biol. Report..

[47] Itoo H., Shah R.A., Qurat S., Jeelani A., Khursheed S., Bhat Z.A., Mir M.A., Rather G.H., Zargar S.M., Shah M.D. (2023). Genome-Wide Characterization and Development of SSR Markers for Genetic Diversity Analysis in Northwestern Himalayas Walnut (*Juglans regia* L.). 3 Biotech.

[48] Orhan E., Eyduran S.P., Poljuha D., Akin M., Weber T., Ercisli S. (2020). Genetic Diversity Detection of Seed-Propagated Walnut (*Juglans regia* L.) Germplasm from Eastern Anatolia Using SSR Markers. Folia Hortic..

[49] Vahdati K., Mohseni Pourtaklu S., Karimi R., Barzehkar R., Amiri R., Mozaffari M., Woeste K. (2015). Genetic Diversity and Gene Flow of Some Persian Walnut Populations in Southeast of Iran Revealed by SSR Markers. Plant Syst. Evol..

[50] Mahmoodi R., Rahmani F., Rezaee R. (2013). Genetic Diversity among *Juglans regia* L. Genotypes Assessed by Morphological Traits and Microsatellite Markers. Span. J. Agric. Res..

[51] Ebrahimi A., Fatahi R., Zamani Z. (2011). Analysis of Genetic Diversity among Some Persian Walnut Genotypes (*Juglans regia* L.) Using Morphological Traits and SSRs Markers. Sci. Hortic..

[52] Feng X., Zhou H., Zulfiqar S., Luo X., Hu Y., Feng L., Malvolti M.E., Woeste K., Zhao P. (2018). The Phytogeographic History of Common Walnut in China. Front. Plant Sci..

[53] Eser E., Topçu H., Kefayati S., Sütyemez M., Islam M.R., Kafkas S. (2019). Highly Polymorphic Novel Simple Sequence Repeat Markers from Class I Repeats in Walnut (*Juglansregia* L.). Turk. J. Agric. For..

[54] Zhou H., Zhao P., Woeste K., Zhang S. (2021). Gene Flow among Wild and Cultivated Common Walnut (*Juglans regia*) Trees in the Qinling Mountains Revealed by Microsatellite Markers. J. For. Res..

[55] Bujdoso G., Cseke K. (2021). The Persian (English) Walnut (*Juglans regia* L.) Assortment of Hungary: Nut Characteristics and Origin. Sci. Hortic..

[56] Aradhya M., Velasco D., Preece J., Kluepfel D.A. (2021). Biogeographic and Glacial History of Walnut (*Juglans regia* L.). Acta Hortic..

[57] Pollegioni P., Woeste K., Chiocchini F., Del Lungo S., Ciolfi M., Olimpieri I., Tortolano V., Clark J., Hemery G.E., Mapelli S. (2017). Rethinking the History of Common Walnut (*Juglans regia* L.) in Europe: Its Origins and Human Interactions. PLoS ONE.

[58] Guney M., Kafkas S., Keles H., Zarifikhosroshahi M., Gundesli M.A., Ercisli S., Necas T., Bujdoso G. (2021). Genetic Diversity among Some Walnut (*Juglans regia* L.) Genotypes by SSR Markers. Sustainability.

[59] Karimi H.R., Mirdehghan S.H. (2013). Correlation between the Morphological Characters of Pomegranate (*Punica granatum*) Traits and Their Implications for Breeding. Turk. J. Bot..

[60] Waits L.P., Luikart G., Taberlet P. (2001). Estimating the Probability of Identity among Genotypes in Natural Populations: Cautions and Guidelines. Mol. Ecol..

[61] Bernard A., Barreneche T., Lheureux F., Dirlewanger E. (2018). Analysis of Genetic Diversity and Structure in a Worldwide Walnut (*Juglans regia* L.) Germplasm Using SSR Markers. PLoS ONE.

[62] Fatahi R., Ebrahimi A., Zamani Z. (2010). Characterization of some Iranians and foreign walnut genotypes using morphological traits and RAPD markers. Hortic Environ. Biotechnol..

[63] Kalinowski S.T., Taper M.L., Marshall T.C. (2007). Revising How the Computer Program cervus Accommodates Genotyping Error Increases Success in Paternity Assignment. Mol. Ecol..

[64] Peakall R., Smouse P.E. (2006). Genalex 6: Genetic Analysis in Excel. Population Genetic Software for Teaching and Research. Mol. Ecol. Notes.

[65] Peakall R., Smouse P.E. (2012). GenAlEx 6.5: Genetic Analysis in Excel. Population Genetic Software for Teaching and Research—An Update. Bioinformatics.

[66] Sneath P.H.A., Sokal R.R. (1973). Numerical Taxonomy: The Principles and Practice of Numerical Classification.

[67] Kumar S., Stecher G., Li M., Knyaz C., Tamura K. (2018). MEGA X: Molecular Evolutionary Genetics Analysis across Computing Platforms. Mol. Biol. Evol..

[68] Pritchard J.K., Stephens M., Donnelly P. (2000). Inference of Population Structure Using Multilocus Genotype Data. Genetics.

[69] Falush D., Stephens M., Pritchard J.K. (2003). Inference of Population Structure Using Multilocus Genotype Data: Linked Loci and Correlated Allele Frequencies. Genetics.

[70] Earl D.A., vonHoldt B.M. (2012). STRUCTURE HARVESTER: A Website and Program for Visualizing STRUCTURE Output and Implementing the Evanno Method. Conserv. Genet. Resour..

