# Supplementary material for: Molecular Characterization of Local Walnut (Juglans regia) Genotypes in the North-East Parnon Mountain Region of Greece
Source: Int J Mol Sci. 2023 Dec 7;24(24):17230. doi: 10.3390/ijms242417230 (PMC10743642; doi:10.3390/ijms242417230)
Supplement: Supplementary file 1 [file ijms-24-17230-s001.zip › ijms-2721686-supplementary.pdf]

Table S1. Alleles frequencies for each SSR marker and all 47 walnut accessions

SSR marker	Allele	Frequency
WGA009	233	0.011
	234	0.021
	235	0.021
	236	0.117
	237	0.011
	238	0.096
	239	0.011
	240	0.106
	241	0.074
	242	0.011
	243	0.053
	244	0.053
	246	0.064
	248	0.043
	263	0.011
	264	0.011
	268	0.043
	269	0.021
	270	0.021
	271	0.043
	273	0.011
	274	0.043
	275	0.011
	276	0.021
	277	0.043
	278	0.011
	279	0.021
WGA321	217	0.011
	219	0.043
	220	0.021
	222	0.053
	223	0.053
	224	0.096
	225	0.074
	226	0.021
	227	0.032
	230	0.032
	231	0.011
	232	0.064
	233	0.043
	234	0.053
	235	0.053
	236	0.096
	237	0.043

Table S1. Alleles frequencies for each SSR marker and all 47 walnut accessions

	238	0.053
	239	0.032
	240	0.043
	241	0.032
	243	0.032
	244	0.011
WGA349	240	0.011
	250	0.021
	251	0.021
	255	0.032
	256	0.085
	257	0.064
	258	0.032
	259	0.043
	260	0.085
	261	0.021
	262	0.085
	263	0.096
	264	0.064
	265	0.074
	266	0.074
	267	0.021
	269	0.043
	270	0.021
	271	0.011
	273	0.043
	274	0.011
	275	0.011
	276	0.021
	277	0.011
WGA069	158	0.021
	159	0.053
	160	0.085
	161	0.096
	162	0.032
	163	0.074
	164	0.074
	165	0.064
	166	0.064
	167	0.053
	168	0.011
	170	0.021
	171	0.032
	172	0.043
	173	0.032

Table S1. Alleles frequencies for each SSR marker and all 47 walnut accessions

	174	0.032
	175	0.032
	176	0.011
	177	0.032
	178	0.021
	179	0.011
	180	0.053
	184	0.011
	185	0.021
	200	0.021
WGA118	184	0.021
	187	0.011
	188	0.043
	189	0.085
	190	0.021
	191	0.021
	192	0.128
	193	0.106
	194	0.064
	195	0.043
	196	0.085
	197	0.085
	198	0.085
	199	0.021
	200	0.043
	201	0.021
	202	0.053
	203	0.021
	207	0.043
WGA202	216	0.021
	221	0.021
	240	0.021
	246	0.106
	247	0.043
	248	0.043
	250	0.064
	252	0.043
	253	0.043
	254	0.064
	255	0.043
	257	0.043
	258	0.021
	259	0.064
	260	0.085
	261	0.021

Table S1. Alleles frequencies for each SSR marker and all 47 walnut accessions

	262	0.085
	265	0.043
	266	0.021
	267	0.021
	269	0.021
	270	0.021
	272	0.021
	273	0.021
WGA276	168	0.011
	170	0.021
	172	0.021
	174	0.032
	175	0.043
	176	0.032
	177	0.043
	178	0.117
	179	0.064
	180	0.064
	181	0.021
	182	0.053
	183	0.032
	184	0.117
	185	0.074
	186	0.021
	187	0.021
	188	0.043
	189	0.064
	190	0.053
	192	0.011
	196	0.011
	198	0.021
	205	0.011
WGA001	185	0.011
	187	0.032
	188	0.011
	189	0.011
	190	0.021
	193	0.011
	195	0.021
	196	0.032
	197	0.043
	198	0.011
	199	0.032
	200	0.021
	201	0.032

Table S1. Alleles frequencies for each SSR marker and all 47 walnut accessions

	202	0.053
	203	0.021
	204	0.011
	205	0.043
	207	0.021
	211	0.011
	214	0.011
	217	0.032
	218	0.043
	219	0.032
	220	0.011
	221	0.043
	222	0.043
	223	0.011
	224	0.053
	226	0.011
	227	0.032
	228	0.032
	229	0.011
	232	0.011
	233	0.021
	234	0.011
	236	0.021
	238	0.021
	239	0.021
	240	0.011
	241	0.011
	242	0.043
	244	0.021
WGA376	218	0.011
	220	0.011
	227	0.021
	230	0.032
	232	0.011
	233	0.053
	234	0.021
	235	0.064
	236	0.043
	237	0.043
	238	0.021
	240	0.032
	241	0.032
	242	0.011
	243	0.064
	244	0.032

Table S1. Alleles frequencies for each SSR marker and all 47 walnut accessions

245	0.011
246	0.021
247	0.021
249	0.074
250	0.011
252	0.064
253	0.011
254	0.064
255	0.032
256	0.043
257	0.043
258	0.011
259	0.011
261	0.043
262	0.011
263	0.011
265	0.011
276	0.011
